# Propiece IL-1α facilitates the growth of acute T-lymphocytic leukemia cells through the activation of NF-κB and SP1

**DOI:** 10.18632/oncotarget.14934

**Published:** 2017-02-01

**Authors:** Yinsheng Zhang, Xiao Yu, Dandan Lin, Lei Lei, Bo Hu, Fengzhang Cao, Yu Mei, Depei Wu, Haiyan Liu

**Affiliations:** ^1^ Institute of Blood and Marrow Transplantation, Jiangsu Institute of Hematology, Collaborative Innovation Center of Hematology, The First Affiliated Hospital of Soochow University, Suzhou 215006, P. R. China; ^2^ Immunology Programme, Life Sciences Institute and Department of Microbiology and Immunology, National University of Singapore, Singapore 117456, Singapore

**Keywords:** propeice IL-1α, acute lymphocytic leukemia, NF-κB, Sp1

## Abstract

Interleukin 1α (IL-1α) is a pro-inflammatory cytokine that possesses multiple immune-regulatory functions. It is mainly expressed as the cell-associated form and not actively secreted in healthy tissues. The intracellular IL-1α has been shown to be a chromatin-associated cytokine and can affect transcription. There are spontaneous expressions of IL-1α in acute lymphocytic leukemia (ALL) blasts. However, the role of nuclear-localized IL-1α in ALL is not clear. Here we showed that overexpression of the nuclear form of IL-1α (propiece IL-1α) could promote proliferation and reduce apoptosis of T-ALL cells. It also increased the ALL cells’ resistance to low serum concentration and cisplatin treatment. *In vivo* growth of the T-ALL cells overexpressing the propiece IL-1α were also enhanced compared to the control cells. Microarray analysis revealed many changes in gene expressions related to cell growth and stress, including a group of metallothionein genes. Moreover, the expressions of transcription factors, NFκB and specific protein 1 (SP1), were up-regulated by propiece IL-1α. Propiece IL-1α could bind to the promoter of SP1 and a binding sequence logo was identified. Therefore, nuclear expression of propiece IL-1α can facilitate the growth of T-ALL cells possibly through the activation of NFκB and SP1.

## INTRODUCTION

Interleukin 1α (IL-1α), like interleukin 1β (IL-1β), is an important regulator during inflammatory and immune responses, angiogenesis and hematopoiesis [1, 2]. Both IL-1α and IL-1β are expressed as precursors that are further processed by proteases into mature secreted forms [3–5]. IL-1β mainly functions through its mature form. However, unlike IL-1β, IL-1α can function as cell-associated forms and is not actively secreted in healthy tissues. The IL-1α precursor contains an active nuclear localization signal (NLS) in its N-terminal propiece [6]. It can be cleaved by calpain to generate secreted IL-1α and the NLS containing IL-1α propiece [7]. IL-1α can be observed in the nucleus in many different cell types, including activated macrophages, keratinocytes, and Chinese hamster ovary (CHO) cells [8]. The presence of IL-1α in the nucleus has linked to its possible role in affecting transcription and regulating cell growth.

IL-1α was found to affect the cell cycle of osteosarcoma cell to reduce cell growth [9]. On the other hand, overexpression of the N-terminal propiece was found to induce malignant transformation and increase invasiveness in a mouse tumor model [10]. The precursor IL-1α can bind to HCLS1-asscoaited factor-1 (Hax-1), which facilities its nuclear translocation in systemic sclerosis fibroblasts [11]. The N-terminal propiece was found to interact with spliceosome complexes and induce apoptosis in malignant cells [12]. IL-1α can also bind to histone acetyltransferase proteins p300, Gcn5 and PCAF in the nucleus [13, 14]. The chromatin-bound IL-1α was found in both melanoma cells and macrophages [15, 16]. The differential release of chromatin-bound IL-1α was suggested to discriminate between necrotic and apoptotic cell death. Although the DNA binding ability of IL-1α has been shown in many studies, the physiological roles of the nuclear IL-1α in different cell types remain unclear. In addition, how the binding of IL-1α to the chromatin affects transcriptions needs further investigation.

The study of the role of IL-1α in tumor development has been focused on the secreted IL-1α. In diethylnitrosamine (DEN) model of liver carcinogenesis, IL-1α released from necrotic hepatocytes initiated inflammatory responses and stimulated compensatory proliferation in carcinogenesis. Interference with IL-1α signaling or ablation of IL-1R1 inhibited hepatocellular carcinoma (HCC) development [17]. On the other hand, fibrosarcoma cells expressing IL-1α in the cytosol and on the cell membrane lost their tumorigenesis, suggesting cell-associated IL-1α could stimulate anti-tumor immune responses [18, 19]. The role of nuclear IL-1α in malignancy has not been studied extensively. IL-1α has been shown to be constitutively produced by adult T-cell leukemia cells and by HTLV-1-transformed cell lines [20, 21]. Acute lymphocytic leukemia (ALL) cells were reported not to express IL-1β [22]. Neutralization of IL-1α could inhibit the spontaneous proliferation of ALL blasts *in vitro* [21]. IL-1 also stimulated the proliferation of ALL cells *in vitro*, which could be inhibited by neutralizing antibodies to GM-CSF, TNFα, IL-3, or IL-6. However, the role of the intracellular IL-1α, especially the nuclear expression of IL-1α in ALL has not been elucidated.

In the current study, we overexpressed the NLS-containing N-terminal propiece of IL-1α in both murine and human T-ALL cells. Propiece IL-1α promoted the proliferation and reduced apoptosis of T-ALL cells. *In vivo* growth of the T-ALL cells overexpressing the propiece IL-1α were also enhanced compared to the control cells in two murine tumor models. Microarray analysis revealed many changes in gene expressions related to cell growth and stress, including a group of metallothionein (MT)genes. Moreover, the expressions of transcription factors, NFκB and SP1, were up-regulated by propiece IL-1α. Propiece IL-1α could bind to the promoter of SP1 through a binding sequence logo. Therefore, nuclear expression of propiece IL-1α can facilitate the growth of T-ALL cells possibly through the activation of NFκB and SP1.

## RESULTS

### Overexpression of IL-1α propiece in the nuclei of T-ALL cells

**Figure 1:**
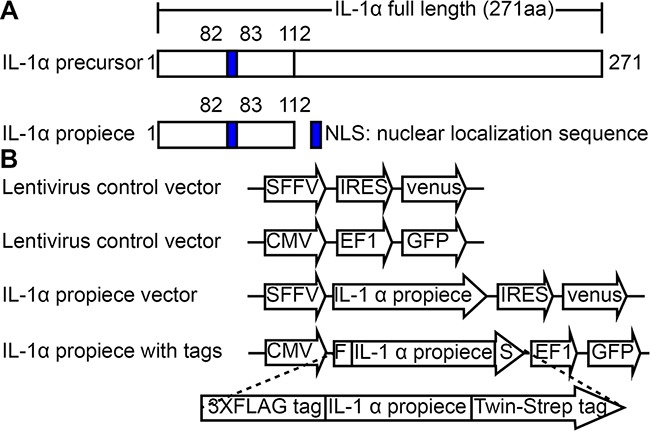
IL-1α propiece expression constructs **A**. schematic representation of human IL-1α full length and propiece sequence. **B**. lentiviral expression vectors expressing IL-1α propiece and IL-1α propiece with triple FLAG tag at 5’ end of ORF and twin strep tag at 3’ end of ORF.

The IL-1α propiece was cloned from human peripheral blood mononuclear cells which included N-terminal 1 to 112 bp (Figure 1A). The NLS sequence is located in that region. The construct was cloned into a lentiviral vector expressing venus. Since there is no antibody available to specifically detect IL-1α propiece, the FLAG tag and strep tag were added to the N-terminal and C-terminal of the construct respectively for detection purpose (Figure 1B).

**Figure 2:**
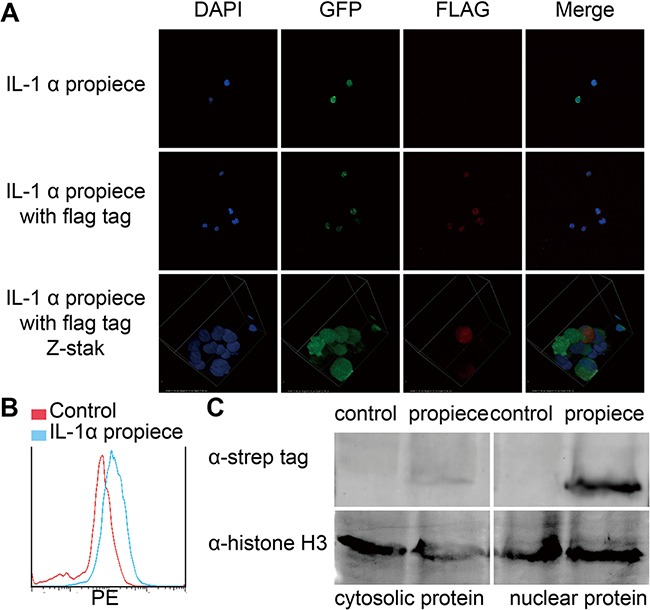
IL-1α propiece is located in nuclei **A**. Jurkat-proIL-1α with FLAG tag (red) was analyzed by immunofluorescence and confocal microscopy (40X). Nuclear DNA was visualized by DAPI staining (blue). **B**. The nuclei of the Jurkat-proIL-1α was analyzed by flow cytometry. **C**. Jurkat-proIL-1α were separated for cytosolic and nuclear proteins, which were then blotted for IL-1α propiece with anti-strep antibodies. Data shown are the representatives of at least three independent experiments.

Jurkat cells stably expressing both constructs were generated by lentiviral infection and fluorescent expression (Figure 2). IL-1α propiece expression was concentrated in the nucleus of the cell by anti-FLAG staining and confocal microscopy (Figure 2A). To further confirm the result, the nuclei were obtained according to the published protocol [23, 24] then analyzed by flow cytometry (Figure 2B). The IL-1α expression was clearly increased in the nuclei of the cells overexpressing IL-1α propiece. The cytosolic and nuclear proteins of Jurkat cells overexpressing IL-1α propiece (Jurkat-proIL-1α) were separated and blotted for strep tag (Figure 2C). The IL-1α propiece was found to be mainly located in the nuclei of the cells. There was a weak signal of IL-1α propiece in the cytoplasm, which might be caused by protein translation in the cytosol or nuclear protein contamination. These results demonstrated that we have overexpressed IL-1α propiece in the nuclei of the T-ALL cells.

### IL-1α propiece promotes proliferation and reduces apoptosis in T-ALL cells *in vitro*

**Figure 3:**
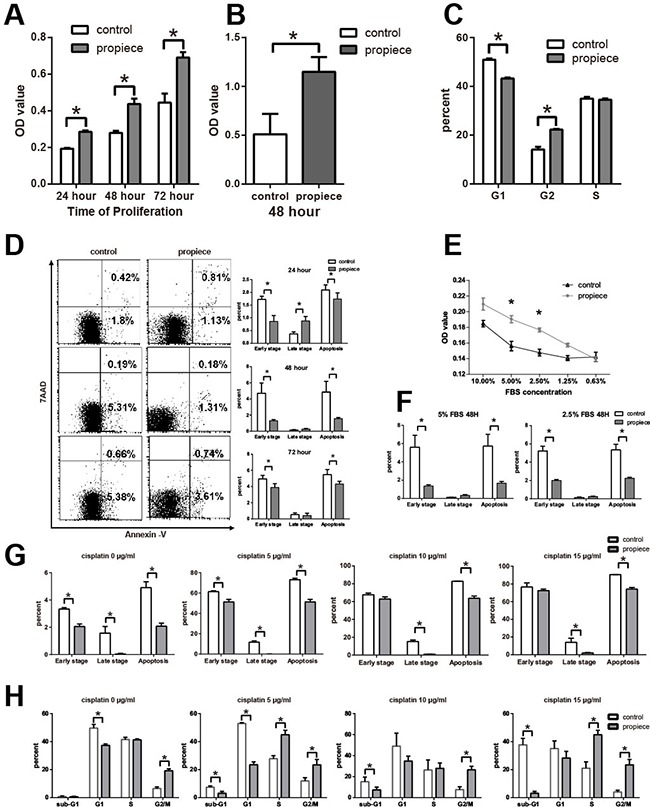
IL-1α propiece promotes proliferation and reduces apoptosis in T-ALL *in vitro* **A**. The proliferations of Jurkat cells were examined by MTT assay at 24, 48 and 72 hours. **B**. The proliferations of p388 cells were examined by MTT assay at 48 hours. **C**. Jurkat-proIL-1α or the control cells were labelled with PI and the percentages of G1, S and G2/M cells were determined by flow cytometry. **D**. Jurkat cells were collected and stained for annexin V and 7AAD. The percentages of apoptotic cells that were labelled with annexin- V^+^ 7AAD^-^ (early stage), annexin- V^+^ 7AAD^+^ (late stage) and both populations (apoptosis) were presented. **E**. Jurkat cells were cultured in decreased FBS concentrations and MTT assays were performed to assess the proliferation of the cells. **F**. Jurkat cells that cultured in 5% and 2.5% FBS concentrations were collected and stained for annexin V and 7AAD. **G**. Jurkat cells that cultured in cisplatin (0, 5, 10, 15 ug/ml) were collected and stained for annexin V and 7AAD. **H**. Jurkat cells cultured in cisplatin were labelled with PI and the percentages of G1, S and G2/M cells were determined by flow cytometry. The experiments were performed with eight samples per group. Data shown are the representatives of at least three independent experiments. Results are expressed as mean ± SD. * *p*<0.05.

To analyze the functional role of IL-1α propiece in T-ALL cells, we performed MTT assays at 24, 48, and 72 hours and Jurkat-proIL-1α cells had increased proliferation at all time points compared to the control cells (Figure 3A). When we overexpressed the murine IL-1α propiece in a murine T-ALL cell line, p388, the proliferation was also increased at 48 hours (Figure 3B). There was also an increase of the cells in G2 phase in Jurkat-proIL-1α cells (Figure 3C), suggesting a shift towards proliferation.

Apoptosis was analyzed by flow cytometry. Both early-stage apoptosis and total apoptosis were reduced in Jurkat-proIL-1α cells at all time points, except increased late-stage apoptosis at 48 hours (Figure 3D). In order to assess whether IL-1α propiece could increase the resistance of the cells to stress, cells were cultured with various low serum concentrations (Figure 3E, 3F). Jurkat-proIL-1α cells survived significantly better than the control cells, suggesting that the IL-1α propiece could increase the cells’ survival under stress (Figure 3E). Early stage apoptosis was significantly reduced in Jurkat-proIL-1α under low serum conditions (Figure 3F). Cisplatin was a platinum-containing anti-cancer chemotherapy drug through G1 phase arrest. Jurkat-proIL-1α cells exhibited less apoptosis under cisplatin treatment compared with the control cells (Figure 3G). Furthermore, IL-1α propiece expression facilitated the resistance to G1 arrest caused by cisplatin (Figure 3H). These *in vitro* results demonstrated that IL-1α propiece could promote proliferation and reduce apoptosis in T-ALL cells.

### IL-1α propiece promotes *in vivo* development of T-ALL

**Figure 4:**
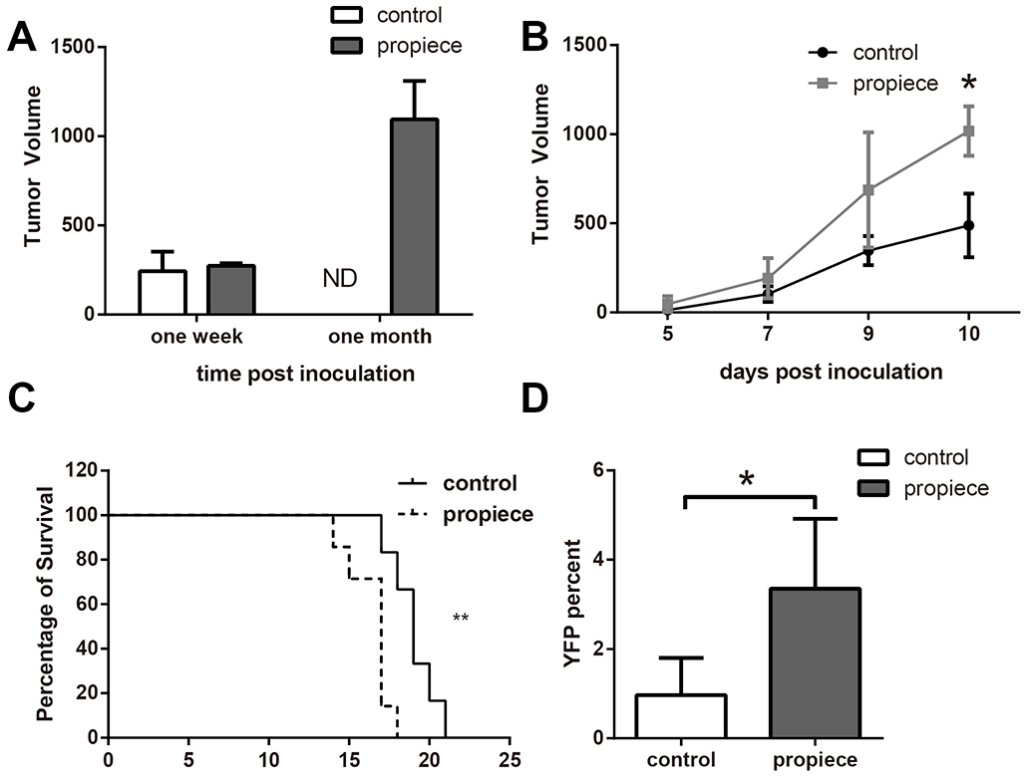
IL-1α propiece promotes the *in vivo* development of T-ALL **A**. Five million Jurkat cells in 100 μl of BD Matrigel were inoculated into nude mice subcutaneously. Tumor sizes were measured at one week and one month after inoculation. **B**. Murine p388 cells (10^5^) were inoculated subcutaneously into DBA mice. Tumor sizes were measured every other day in a blinded fashion. **C**. DBA mice inoculated with p388 cells subcutaneously developed metastatic leukemia. The survival curves of the mice that received p388 cells expressing IL-1α propiece or the control vector were plotted. Statistical differences in survival times were determined using Kaplan–Meier survival curves and X ^2^ analysis. **D**. The YFP positive cells in peripheral blood of the p388 tumor-bearing mice were analyzed by flow cytometry. The experiments were performed with five mice per group. Data shown are the representatives of at least three independent experiments. Results are expressed as mean ± SD. * *p*<0.05, **p<0.01.

In order to confirm the role of IL-1α propiece in T-ALL, we inoculated Jurkat-proIL-1α cells and the control cells subcutaneously in the immune-deficient mice (Figure 4A). The nodule sizes were similar at one week. Then, the control tumors regressed to be undetectable afterwards, while the tumors expressing IL-1α propiece remained in large size by one month (Figure 4A).

A murine T-ALL model was utilized to confirm the *in vivo* role of IL-1α propiece in T-ALL (Figure 4B-D). The murine T-ALL p388 cells were injected into immune-competent mice subcutaneously and the tumor growth was monitored (Figure 4B). The tumors grew at the similar rate initially, but the tumors expressing IL-1α propiece were significantly larger than the control tumors by day 10. The tumors then developed to metastatic leukemia and caused death of the tumor-bearing mice (Figure 4C). The mice received p388 cells expressing IL-1α propiece had accelerated death compared with the mice received control cells, suggesting that IL-1α propiece promoted the development of metastatic T-ALL. Since the transfected p388 cells expressed yellow fluorescent protein (YFP, venus), the percent of cells expressing YFP was detected from the peripheral blood of the leukemia-bearing mice (Figure 4D). The mice received p388 cells expressing IL-1α propiece had significantly higher percentages of YFP positive cells in the peripheral blood, demonstrating a higher load of leukemia cells. Therefore, the IL-1α propiece could promote the development of T-ALL *in vivo*.

### IL-1α propiece can activate NFκB and SP1

**Figure 5:**
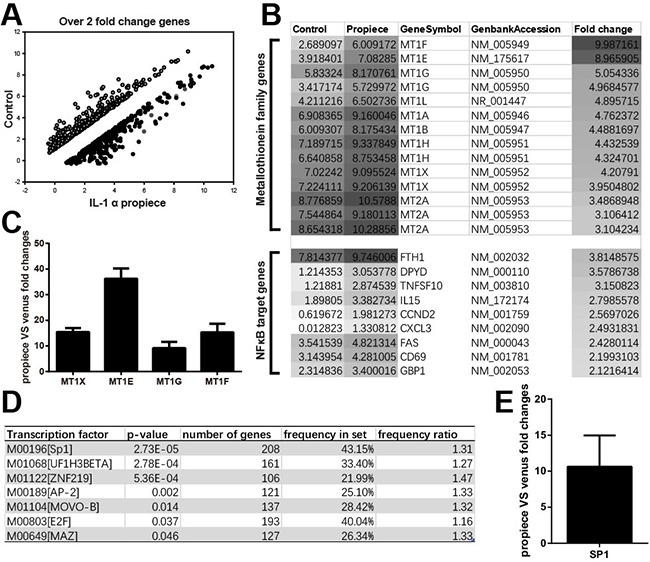
Microarray and real-time PCR analysis of the gene expressions in Jurkat-proIL-1α and control cells **A**. Whole Genome Oligo Array (4×44K) was performed. The gene expressions were compared between IL-1α propiece and control group. **B**. Enrichment analysis of genes expressions in Jurkat cells, MT family genes and NFkB target genes were up-regulated. **C**. Quantitative PCR analysis of the MT gene expressions in Jurkat-proIL-1α cells. **D**. Enrichment analysis for related promoters aiming to find affected transcription factors by PRIMA (included in Expander5.2). *p*-value was set at 1E-4 and only SP1 promoter was affected. **E**. Quantitative PCR analysis of SP1 expression in Jurkat-proIL-1α cells. The Quantitative PCR (qPCR) analysis were performed in five replicates. Data shown are the representatives of at least three independent experiments. Results are expressed as mean ± SD.

To investigate the possible mechanism of IL-1α propiece regulating proliferation, we performed microarray analysis to compare the gene expression profiles between the Jurkat-proIL-1α and the control cells (Figure 5A). Among the genes that were up-regulated by IL-1α propiece expression, there was a group of MT genes and NFκB target genes that were dramatically increased (Figure 5B). MT genes include MT1X, MT1E, MT1G, and MT1F and they were also confirmed by real-time PCR (Figure 5C). Moreover, transcription factor analysis revealed a group of transcriptions factors that could be regulated by IL-1α propiece to affect down-stream gene expressions and SP1 exhibited the highest significance (Figure 5D). The upregulation of SP1 expression by IL-1α propiece was also confirmed by real-time PCR (Figure 5E).

**Figure 6:**
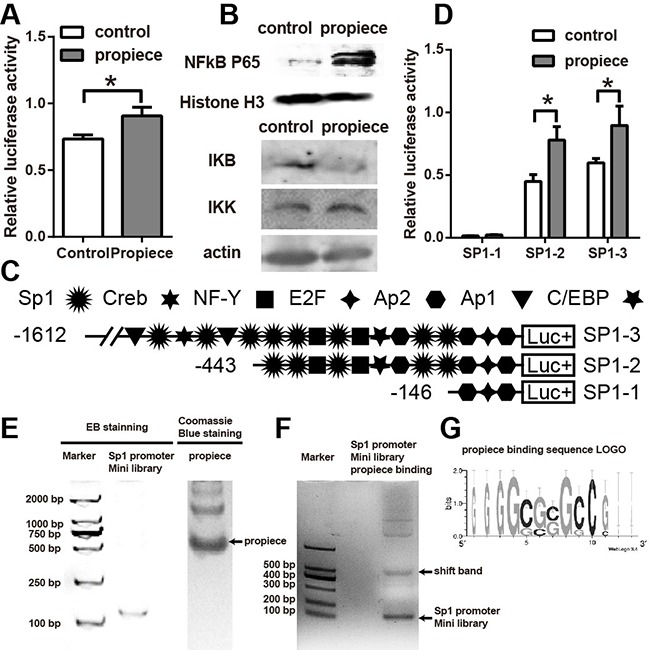
NFκB and SP1 are activated by IL-1α propiece expression **A**. NFκB promoter reporter assay in 293T cells expressing IL-1α propiece or the control vector. **B**. Nuclear proteins were extracted from Jurkat-proIL-1α or the control cells and blotted for p65 and Histone H3. Cytoplasmic proteins were also extracted and blotted for IκB and IKK expressions. **C**. Schematic illustration of the SP1 promoter deletion constructs. The locations of putative nuclear protein binding sites are indicated. **D**. Each construct was transiently transfected into 293T cells and assayed for luciferase activity. **E**. SP1 promoter mini library was constructed by 80bp fragments with 14bp overlapping that cover 1612 bp of SP1 promoter. The SP1 promoter mini library was run on a PAGE gel then stained with Ethidium Bromide (EB). Purified IL-1α propiece was run on a PAGE gel then stained with coomassie blue. **F**. In-gel EMSA was performed by using IL-1α propiece and SP1 promoter mini library. **G**. High affinity IL-1α propiece-binding DNA fragments obtained from SELEX experiments were sequenced and analyzed by WebLogo 3.4 for binding sequence LOGO. The assays were performed in quadruplicates. Data shown are the representatives of at least three independent experiments. Results are expressed as mean ± SD. * *p*<0.05.

We then performed luciferase reporter assay with the NFκB promoter (Figure 6A). The data showed that the cells overexpressing IL-1α propiece had increased NFκB promoter activity compared with the control cells. Moreover, there was also an increased expression of NFκB p65 in the nuclei and a decreased expression of IκB in the cytoplasm of the cells overexpressing IL-1α propiece (Figure 6B). The luciferase reporter assays were also performed with the SP1 promoter (Figures 6C and 6D). The SP1 promoter was expressed in different lengths in order to identify the possible binding region of IL-1α propiece (Figure 6C). SP1-3 had significantly higher promoter activity in the cells overexpressing IL-1α propiece, suggesting IL-1α propiece could promote SP1 activation. When the region between 443 and 1612 bp were truncated, SP1-2 still showed increased promoter activity. Furthermore, the promoter activity was diminished in both cells overexpressing IL-1α propiece and the control cells when SP1-1 (only 146 bp remained) was used in the assay. These results suggested that IL-1α propiece could regulate the promoter activity of SP1 through the region between 146 and 443 bp. The SELEX experiments were performed after SP1 mini library was constructed and IL-1α propiece was purified (Figure 6E). A band shift could be detected by in-gel electrophoretic mobility shift assay (EMSA). The sub DNA library was cloned from the shifted band then another SELEX was preformed using the purified IL-1 α propiece and sub DNA library. The SELEX experiments were repeated four times and the binding fragments were sequenced. A binding sequence LOGO was identified to be GGGGCGCGCCG after sequence alignment and analysis with DNA LOGO application (Figure 6G)

## DISCUSSION

Previous studies have shown that IL-1α can be constitutively produced by adult T cell leukemia and was considered as an autocrine growth factor for ALL [20, 21, 30]. The calpain cleavage is required to generate the secreted IL-1α and at the same time the N-terminal propiece can be formed and translocate to the nuclei. Although there are some studies focusing on the secreted IL-1α, the function of IL-1α propiece in T-ALL has not been elucidated. In the current study, we demonstrated for the first time that overexpression of the IL-1α propiece could promote the proliferation and reduce apoptosis of T-ALL cells. *In vivo* growth of the T-ALL cells overexpressing the propiece IL-1α were also enhanced compared to the control cells. Moreover, the expressions of transcription factors, NFκB and SP1, were up-regulated by propiece IL-1α. We demonstrated that propiece IL-1α could bind to the SP1 promotor region. Therefore, nuclear expression of propiece IL-1α could facilitate the growth of T-ALL cells possibly through the activation of NFκB and SP1.

IL-1α was up-regulated and translocated to the nuclei when the macrophages were stimulated with the viruses [31]. The nuclear translocation of IL-1α was also observed in both murine and human macrophages stimulated with lipopolysaccharide (LPS) [32]. Nuclear IL-1α may also play a role during the apoptosis and senescence in response to 1,25 Dihydroxyvitamin D3 [33, 34]. However, whether the nuclear IL-1α was the full length IL-1α or the propiece IL-1α was not clearly defined in those studies due to the limitation of the detection methods. IL-1α is not readily secreted in healthy tissues. Therefore, the cleavage of the full-length IL-1α may not occur during homeostasis. The nuclear IL-1α may be the full-length IL-1α in that situation. During tumor development, secreted IL-1α was found to be increased and the secreted IL-1α in the tumor microenvironment could lead to exacerbation of tumor growth [8]. Meanwhile propiece IL-1α should be generated by the calpain cleavage and the major nuclear form of IL-1α may be the propiece IL-1α during tumor development [1, 35]. The expression pattern of the nuclear IL-1α and whether the function of the full-length and the propiece IL-1α differ still need further investigation.

The microarray data showed that the overexpression of IL-1α in the nuclei had a profound effect on the transcriptions of the molecules in the proliferation, apoptosis and anti-stress pathways. The expressions of the MT genes were dramatically increased by IL-1α propiece expression (Figure 5B). MT genes can up-regulate the expressions of mitogens, growth factors and inhibit apoptosis [36]. Both E2F7 and SP1 expressions were increased in the cells overexpressing IL-1α propiece (Figure 5C, data not shown), which were important transcription factors regulating MT expressions [37]. SP1 can also promote the activation of NFκB, which is the master regulator during cell proliferation [38, 39]. We confirmed by the luciferase promoter assay that both SP1 and NFκB could be activated by IL-1α propiece overexpression (Figure 6). However, whether the activation of NFκB is dependent on SP1 still needs to be assessed.

IL-1α and IL-1β are important cytokines in local and systemic inflammation. Many strategies blocking their activities have been tested in human inflammation-related diseases [40]. Monoclonal antibody to IL-1α is being tested in cancer, especially leukemia treatment. While the effect of the secreted IL-1α could be blocked by this treatment, our results suggested that the nuclear IL-1α propiece generated during the process of IL-1α secretion could still promote leukemia growth. Therefore, inhibition of the enzymatic process of IL-1α cleavage may be a more effective strategy treating cancer and leukemia in the future.

## MATERIALS AND METHODS

### Cloning of IL-1α propiece and overexpression in Jurkat and p388 cells

Human and mouse IL-1α propiece genes were obtained by PCR from cDNA of human or mouse peripheral blood cells. The gene was ligated into pRRL-Venus lentiviral vector that was kindly provided by Dr. Christopher Baum (Hannover Medical School, Germany). For detection purpose, the triple FLAG tag and twin strep tag were linked to IL-1α propiece and then cloned into SBI CD513b plasmid (System Biosciences (SBI), Mountain View, CA). The primers used for cloning are listed in Supplementary Table 1 and the sequences of the cloned and the recombinant products are included in Supplementary Table 2, which have been confirmed by sequencing.

Lentivirus packaging and harvest: Split the 293T cells in logarithmic growth phase in 10 cm dish (6 million / dish) on day 1. The media was changed to 7 ml 10% DMEM (Gibco/Invitrogen, Carlsbad, CA) on day 2. 2.5 M CaCl_2_ and 2 × HBS were prepared freshly (both from Sigma, St. Louis, MO). The recombinant lentivirus plasmid was mixed with the package plasmid (ΔR, VSV-G, Rev). Then 50μl CaCl_2_ (Sigma, St. Louis, MO) was added dropwisely to DNA solution. Then the mixture was transferred to the same volume 2 × HBS dropwisely, incubated at room temperature for 5 min. 1ml mixture was added to each dish. On day 3, the media was changed with 5 ml 10% DMEM. On day 4, the virus containing media was collected and put through 0.2 μM filter and the virus was concentrated by ultracentrifugation (25,000 rpm / 1.5 h).

Jurkat and p388 cells in logarithmic growth phase were harvested and adjusted to 5×10^5^/ml. Cells were added 3 ml per well to 6-well plate. 1 ml recombinant lentivirus was added per well and mixed gently. The rate of infection was detected by flow cytometry after 72 hours. The brightest 5% YFP positive cells were single-cell sorted by FACS Aria (BD, Franklin Lakes, NJ). The colony was cultured in complete DMEM medium (Sigma, St. Louis, MO) for two to three weeks. The cells were then screened by FACS Canto (BD, Franklin Lakes, NJ) for the YFP expression and purity.

### Immunofluorescent staining and confocal microscopy

After fixation by 4% formaldehyde for 15 min, cells were permeabilized with 0.1% saponin (Sigma, St. Louis, MO) for 15 min. Cells were washed three times in PBS and blocked in blocking buffer (1X PBS/5% normal serum/0.3% Triton™ X-100) for 60 min. Anti-FLAG antibody (1X PBS/1% BSA/0.3% Triton™ X-100) was applied at 1:500 (GenScript, Nanjing, China) and the incubation went overnight at 4 degree. After washing three times with PBS, the secondary PE-conjugated goat anti-mouse antibody (Abcam, Cambridge, UK, 1:500) was applied and incubated for 2 hours at room temperature. The cells were stained with 4′,6-diamidino-2-phenylindole (DAPI) (Sigma, St. Louis, MO) and washed three times with PBS before they were sealed with nail polish and analyzed under a fluorescence confocal microscope (Nikon, Tokyo, Japan).

### Flow cytometry for nuclear protein

The nuclei of Jurkat cells were prepared as described [23, 24]. Briefly, the cells were washed twice with PBS. Then, the cells were resuspended in buffer F1 (20 mM Tris, pH 7.6, 50 mM 2-mercaptoethanol, 0.1 mM EDTA, 2 mM MgCl2). After 2 min of incubation at room temperature and 10 min on ice, detergent IGEPAL CA-630 (Sigma, St. Louis, MO) was added to the concentration of 0.5% and the cells were homogenized by passing through a 20G needle for three times. The nuclei were harvested by centrifugation for 5 min at 600g at 4°C and washed three times in buffer F1 supplemented with 0.05% IGEPAL CA-630. After staining with anti-FLAG antibody (GenScript, Nanjing, China, 1:500) and a secondary PE-conjugated goat anti-mouse antibody (Abcam, Cambridge, UK, 1:500), cells were analyzed using a BD FACSCalibur™ flow cytometer (BD, Franklin Lakes, NJ).

### Separation of cytosolic and nuclear proteins and western blot

Jurkat cells were washed twice with ice-cold PBS. To extract cytosolic and nuclear protein, Nuclear and Cytoplasmic Protein Extraction Kit was used according to the manufacture's instructions (Beyotime, Haimen, China). Proteins from the extraction were resolved by electrophoresis, transferred to a PVDF membrane, and hybridized with antibodies for strep (GenScript, Nanjing, China, 1:2000), H3 (Abcam, Cambridge, UK, 1:4000), actin (GenScript, Nanjing, China, 1:2000), NFκB P65 (Sangon Biotech, Shanghai, China, 1:500), IKK and IκB (Beyotime, Shanghai, China, 1:1000). Next, the membranes were incubated in secondary antibody, IRDye® 800CW Goat anti-Mouse IgG and IRDye® 680RD Goat anti-Rabbit IgG (both from LI-COR Biosciences, Lincoln, NE) for 2 hours and the specific bands on the membranes were detected using Odyssey® Imaging Systems (LI-COR Biosciences, Lincoln, NE).

### 3-(4,5-dimethylthiazol-2-yl)-2,5-diphenyltetrazolium bromide (MTT) assay

2×10^3^ Jurkat cells were cultured in 96-well plate for 24, 48 and 72 hours following Mosmann's protocol [25]. MTT (Sigma, St. Louis, MO) was dissolved in PBS at 5mg/ml and filtered to sterilize. 10μl MTT solution was added per 100 μl medium 4 hours before detection. Acid-isopropanol (100μl of 0.04M HCl in isopropanol) was added to wells and mixed thoroughly. After all crystals were dissolved, the plates were read at wavelength of 570nm on a spectrometer.

### Flow cytometry for apoptosis analysis

1×10^6^ Jurkat cells were harvested and washed with PBS twice. Dilute PE-Annexin V (BioLegend, San Diego, CA) at a concentration of 1 mg/ml in binding buffer (10 mM HEPES/NaOH, pH 7.4, 150 mM NaCl, 5 mM KCl, 1 mM MgCl_2_, 1.8 mM CaCl_2_) and resuspend cells in 100 μl of this solution (prepare it freshly each time). Incubate 10 min in the dark at room temperature. Add to the cell suspension 100 μl 7-aminoactinomycin D (7AAD) (BD Biosciences, Heidelberg, Germany) solution prior to analysis to give a final concentration of 1 mg/ml. Cells were analyzed by a BD Calibur (BD, Franklin Lakes, NJ).

### Cell cycle analysis

Suspend cells at 1–2 ×10^6^ cells per ml in 1 ml PBS. Then centrifuge at 200g for 5 min at room temperature. Aspirate off the PBS. Resuspend cell pellet in 500 μl of PBS then fix cells by adding 4.5 ml of 70% (v/v) cold ethanol. The cell suspension should be kept on ice. Centrifuge at 400g for 5 min and remove the supernatant. Wash cells in 5 ml PBS and centrifuge at 400g for 5 min. Resuspend cells in 500 μl of PBS and add 500 μl DNA extraction buffer. Incubate at room temperature for 5 min and centrifuge at 400g for 5 min. Remove the supernatant and resuspend cells in 1 ml of DNA staining solution. Incubate for at least 30 min at room temperature in the dark. Analyze cell cycle by flow cytometry (BD, Franklin Lakes, NJ).

### Animal models

Female nude mice (BALB/c nu) and male DBA mice (DBA/2) were purchased from Shanghai Laboratory Animal Center (Shanghai, China) and housed in sterile, filter- capped cages on a 12-h light/dark cycle and allowed sterile water and irradiated rodent diet. All the animal experiments were approved by the institutional animal care and use committee of Soochow University. Five million Jurkat cells in 100 μl of BD matrigel (BD, Franklin Lakes, NJ) were injected subcutaneously into the flank region of six-week old mice. Tumor sizes were measured at one week and one month, and tumor volumes were calculated as: V=(π/6)×L×W^2^ (perpendicular length (L) and width (W)). 1×10^5^ p388 cells were injected subcutaneously into the six-week old DBA mice and the tumor sizes were measured and volumes were calculated as above. The survival of the mice was also monitored.

### Microarray and real-time PCR

Total RNA was harvested from Jurkat cells using TRIzol reagent (Invitrogen, Carlsbad, CA) according to the manufacturer's instructions. Total RNA from each sample was quantified using the NanoDrop 1000 (Thermo Fisher Scientific, Waltham, MA) and RNA integrity was assessed using standard denaturing agarose gel electrophoresis. For Agilent Human 4×44K Gene Expression Microarrays analysis, the Agilent Array platform was used employing the manufacturer's standard protocols for sample preparation and microarray hybridization. Briefly, total RNA (1 μg) from each sample was amplified and transcribed into fluorescent cRNA following the manufacturer's Quick Amp Labeling protocol (Version 5.7, Agilent Technologies, Santa Clara, CA). Labelled samples were hybridized towards the Whole Genome Oligo Array (4×44K, Agilent Technologies, Agilent Scanner G2505B, Santa Clara, CA) and following the washing steps the arrays were scanned using the Agilent Scanner G2505B (Agilent Technologies, Santa Clara, CA).

Agilent Feature Extraction Software (version 10.5.1.1) was used to analyze the acquired array images. Median normalization and subsequent data processing were performed using the GeneSpringGX v11.0 software package (Agilent Technologies, Santa Clara, CA). Following median normalization of the raw data, genes that at least 4 out of 4 samples have flags in Present (“All Targets Value”) were chosen for further data analysis. Differentially expressed genes were identified through Fold Change filtering. Pathway analysis and GO Analysis were performed to reveal the biological functions of this subset of differentially expressed genes. Finally, hierarchical clustering was performed to show distinguishable gene expression profiling among samples. Differentially expressed genes were selected and validated by real-time PCR using SYBR Green as described previously [26, 27]. Primers were designed using Beacon Designer software or referenced from the published papers [28, 29].

### Transcription factor analysis

Transcript factor analysis was performed using Expander application. The last version of the Expander can be download at (http://acgt.cs.tau.ac.il/expander/). Expander provides promoter analysis utility by integrating PRIMA (PRomoter Integration in Microarray Analysis) tool. PRIMA is a program for finding transcription factors (TFs) whose binding sites are enriched in a given set of promoters. It is aimed at the identification of TFs that take part in these networks. The data was load in the expander then PRIMA tool was used to process the data.

### Reporter assay for NFκB and SP1 promoter activity

293T cells that expressed IL-1α propiece or venus as control were cultured in 100mm dish to reach 80% confluent. The cells were transfected with 10 ug of pRL renilla luciferase reporter plasmid as internal control and 10ug NFκB luciferase reporter plasmid. At 48 hours post-transfection, the luciferase activity was measured using a dual glow kit (Promega, Fitchburg, WI) according to the manufacturer's instruction. Briefly, discard growth media from cultured cells then wash one time by 1×PBS. Lyse cells for 15 minutes at room temperature by 1×Passive Lysis Buffer (PLB). Transfer 20 μl cell lysate to a new plate and measure the firefly luciferase activity after dispensing 100 μl Luciferase Assay Substrate in Luciferase Assay Buffer II. Then measure Renilla luciferase activity after adding 100μl of Stop & Glo® Reagent.

Progressive deletions were made in the SP1 promoter region and inserted between the NheI and XhoI sites of the reporter luciferase vector pGL3-basic (Promega, Fitchburg, WI). The primers used for constructing the SP1 region are listed in Supplementary Table 3. SP1-1, SP1-2, SP1-3 stand for 1612 bp, 443 bp, 146 bp from the 5’-UTR of the sequence to the translational start site.

### Systematic evolution of ligands by exponential enrichment (SELEX)

IL-1α propiece was prokaryotic expressed and purified by affinity chromatography. The SP1 promoter mini library was constructed by cloning the promoter region into 80bp fragments using the primers listed in Supplementary Table 4 (also shown in Supplementary Figure 1). The overlap region was 14bp. The binding DNA fragments was recovered by QIAGEN II (Qiagen Hilden, Germany) from shift band after SELEX was performed. Repeat the experiment four times, then the high affinity binding fragments were cloned and sequenced. Sequence logo was analyzed by importing the high affinity sequences into the web-based application WebLogo (http://weblogo.threeplusone.com).

### Statistics

All data represents at least three independent experiments and results were shown as mean±SD. Statistical analyses were performed with the Graphpad prism 6 (Graphpad, San Diego, CA). Statistical differences in animal survival curves were analyzed by the log-rank test. Other statistical differences between two groups were determined by Student's t-test. A significant difference was considered as *p*<0.05.

## SUPPLEMENTARY MATERIALS FIGURES AND TABLES







## Abbreviations

ALL: acute lymphocytic leukemia
7AAD: 7-aminoactinomycin D
CHO: Chinese hamster ovary
DAPI: 4′,6-diamidino-2-phenylindole
DEN: diethylnitrosamine
EMSA: electrophoretic mobility shift assay
Hax-1: HCLS1-associated protein X-1
HCC: hepatocellular carcinoma
LPS: lipopolysaccharide
MT: metallothionein
MTT: 3-(4,5-dimethylthiazol-2-yl)-2,5-diphenyl-tetrazolium bromide
NLS: nuclear localization signal
PRIMA: PRomoter Integration in Microarray Analysis
SELEX: systematic evolution of ligands by exponential enrichment
SP1: specificity protein 1
TFs: transcription factors
YFP: yellow fluorescent protein
