# Supplementary material for: Propiece IL-1α facilitates the growth of acute T-lymphocytic leukemia cells through the activation of NF-κB and SP1
Source: Oncotarget. 2017 Feb 1;8(9):15677–88. doi: 10.18632/oncotarget.14934 (PMC5362515; doi:10.18632/oncotarget.14934)
Supplement: Supplementary file 1 [file oncotarget-08-15677-s001.pdf]

Propiece IL-1α facilitates the growth of acute T-lymphocytic leukemia cells through the activation of NF-κB and SP1

SUPPLEMENTARY FIGURE AND TABLES

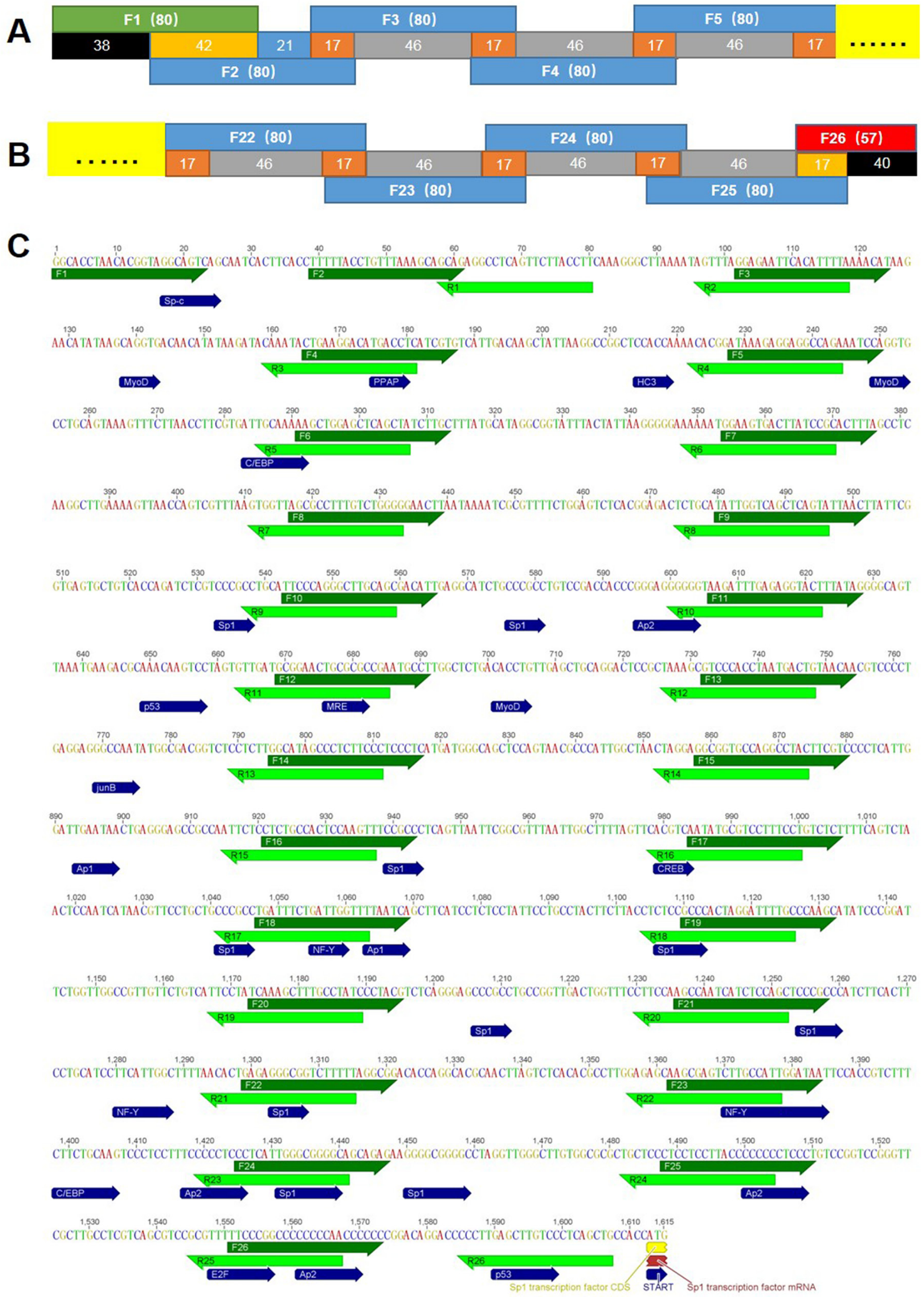

Supplementary Figure 1: Schematic map of SP1 mini library. A. The 5′-end of distribution of the fragments of the mini library. **B.** The 3′-end of distribution of the fragments of the mini library. **C.** The schematic map of all fragments distribution in mini library. Known transcription factor binding sites are also shown.

Supplementary Table 1: Primer sequences for cloning human and murine IL-1α propiece

Primers	Sequence
Forward primer for human IL-1α propiece	GTCGAGCTCGATGGCCAAAGTTCC
Reverse primer for human IL-1α propiece	GTCGAGCTCGATGGCCAAAGTTCC
Forward primer for murine IL-1α propiece	CCGGAATTCCGGCCACCATGGACT
Reverse primer for murine IL-1α propiece	CGCGGATCCGCGTTATTTTTCGAACTGC

Supplementary Table 2: Sequences of cloned and recombinant constructs*.

See Supplementary File 1

Supplementary Table 3: Primer sequences for constructing Sp1 promoter regions

Primers	Sequence
Fwd1:	5′-tcaagtcaggctagcGGGCTTGTGGCGCGCTGCTC-3′
Fwd2:	5′-tcaagtcaggctagcCTATCAAAGCTTTGCCTATCC-3′
Fwd3:	5′-tcaagtcaggctagcGGCACCTAACACGGTAGGCAG-3′
Rev:	5′-cagtgctgcctcgagGCTCAAGGGGGTCCTGTCCGG-3′

Supplementary Table 4: Primer sequences for constructing Sp1 mini library.

See Supplementary File 2
