# Supplementary material for: Propiece IL-1α facilitates the growth of acute T-lymphocytic leukemia cells through the activation of NF-κB and SP1
Source: Oncotarget. 2017 Feb 1;8(9):15677–88. doi: 10.18632/oncotarget.14934 (PMC5362515; doi:10.18632/oncotarget.14934)
Supplement: Supplementary file 2 [file oncotarget-08-15677-s002.docx]

**Supplemental table 2 Sequences of cloned and recombinant constructs*.**

| Construct Name | Sequence |
| --- | --- |
| IL-1α propiece | GTC(GAGCTC)GATGGCCAAAGTTCCAGACATGTTTGAAGACCTGAAGAACTGTTACAGTGAAAATGAAGAAGACAGTTCCTCCATTGATCATCTGTCTCTGAATCAGAAATCCTTCTATCATGTAAGCTATGGCCCACTCCATGAAGGCTGCATGGATCAATCTGTGTCTCTGAGTATCTCTGAAACCTCTAAAACATCCAAGCTTACCTTCAAGGAGAGCATGGTGGTAGTAGCAACCAACGGGAAGGTTCTGAAGAAGAGACGGTTGAGTTTAAGCCAATCCATCACTGATGATGACCTGGAGGCCATCGCCAATGACTCAGAGGAAGAAATCATCAAGCCTAGGCGG[GGTACC]CCG |
| 3×FLAG tag | CCG(GAATTC)CGGCCACCATGGACTACAAAGACCATGACGGTGATTATAAAGATCATGATATCGATTACAAGGATGACGATGACAAGTC<GAGCTC>GATGGCCA |
| Twin Strep tag | AAG[GGTACC]CCGAGCGCTTGGAGCCACCCGCAGTTCGAGAAAGGTGGAGGTTCCGGAGGTGGATCGGGAGGTGGATCGTGGAGCCACCCGCAGTTCGAAAAATAACGC(GGATCC)GCG |
| IL-1α propiece for lentiviral overexpression | CCG{GAATTC}CGGCCACCATGGACTACAAAGACCATGACGGTGATTATAAAGATCATGATATCGATTACAAGGATGACGATGACAAGTC<GAGCTC>GATGGCCAAAGTTCCAGACATGTTTGAAGACCTGAAGAACTGTTACAGTGAAAATGAAGAAGACAGTTCCTCCATTGATCATCTGTCTCTGAATCAGAAATCCTTCTATCATGTAAGCTATGGCCCACTCCATGAAGGCTGCATGGATCAATCTGTGTCTCTGAGTATCTCTGAAACCTCTAAAACATCCAAGCTTACCTTCAAGGAGAGCATGGTGGTAGTAGCAACCAACGGGAAGGTTCTGAAGAAGAGACGGTTGAGTTTAAGCCAATCCATCACTGATGATGACCTGGAGGCCATCGCCAATGACTCAGAGGAAGAAATCATCAAGCCTAGGCGG[GGTACC]CCGAGCGCTTGGAGCCACCCGCAGTTCGAGAAAGGTGGAGGTTCCGGAGGTGGATCGGGAGGTGGATCGTGGAGCCACCCGCAGTTCGAAAAATAACGC{GGATCC}GCG |
| Murine IL-1α propiece for lentiviral overexpression | {GGATCC}ATGGCCAAAGTTCCTGACTTGTTTGAAGACCTAAAGAACTGTTACAGTGAAAACGAAGACTACAGTTCTGCCATTGACCATCTCTCTCTGAATCAGAAATCCTTCTATGATGCAAGCTATGGCTCACTTCATGAGACTTGCACAGATCAGTTTGTATCTCTGAGAACCTCTGAAACGTCAAAGATGTCCAACTTCACCTTCAAGGAGAGCCGGGTGACAGTATCAGCAACGTCAAGCAACGGGAAGATTCTGAAGAAGAGACGGCTGAGTTTCAGTGAGACCTTCACTGAAGATGACCTGCAGTCCATAACCCATGATCTGGAAGAGACGTAA {GGATCC} |

*Endonuclease sites were show in yellow highlights (EcoRI(GAATTC), SacI<GAGCTC>, KpnI[GGTACC] and BamHI{GGATCC}). The 3×FLAG tag sequence was labeled with straight underline. The Twin Strep tag sequence was labeled with wavy underline. IL-1α propiece sequence was indicated with double straight underline.
