# Supplementary material for: Propiece IL-1α facilitates the growth of acute T-lymphocytic leukemia cells through the activation of NF-κB and SP1
Source: Oncotarget. 2017 Feb 1;8(9):15677–88. doi: 10.18632/oncotarget.14934 (PMC5362515; doi:10.18632/oncotarget.14934)
Supplement: Supplementary file 3 [file oncotarget-08-15677-s003.docx]

**Supplemental table 4 Primer sequences for constructing Sp1 mini library.**

| Primer No. | Sequences |
| --- | --- |
| F01 | TAGGGAAGAGAAGGACATATGATGGCACCTAACACGGTAGGCAGTC |
| F02 | TAGGGAAGAGAAGGACATATGATTTTTTACCTGTTTAAAGCAGCAG |
| F03 | TAGGGAAGAGAAGGACATATGATGGAGAATTCACATTTTAAAACAT |
| F04 | TAGGGAAGAGAAGGACATATGATCTGAAGGACATGACCTCATCGTG |
| F05 | TAGGGAAGAGAAGGACATATGATATAAAGAGGAGGCCAGAAATCCA |
| F06 | TAGGGAAGAGAAGGACATATGATAAGCTGGAGCTCAGCTATCTTGC |
| F07 | TAGGGAAGAGAAGGACATATGATGGAAGTGACTTATCCGCACTTTA |
| F08 | TAGGGAAGAGAAGGACATATGATAGCGCCTTTGTCTGGGGGAACTT |
| F09 | TAGGGAAGAGAAGGACATATGATTATTGGTCAGCTCAGTATTAACT |
| F10 | TAGGGAAGAGAAGGACATATGATTTCCCAGGGCTTGCAGCGACATT |
| F11 | TAGGGAAGAGAAGGACATATGATAAGATTTGAGAGGTACTTTATAG |
| F12 | TAGGGAAGAGAAGGACATATGATGCGGAACTGCGCGCCGAATGCCT |
| F13 | TAGGGAAGAGAAGGACATATGATGTCCCACCTAATGACTGTAACAA |
| F14 | TAGGGAAGAGAAGGACATATGATGGCATAGCCCTCTTCCCTCCCTC |
| F15 | TAGGGAAGAGAAGGACATATGATGGCGGTGCCAGGCCTACTTCGTC |
| F16 | TAGGGAAGAGAAGGACATATGATCTCTGCCACTCCAAGTTTCCGCC |
| F17 | TAGGGAAGAGAAGGACATATGATAATATGCGTCCTTTCCTGTCTCT |
| F18 | TAGGGAAGAGAAGGACATATGATTGATTTCTGATTGGTTTTAATCA |
| F19 | TAGGGAAGAGAAGGACATATGATGCCCACTAGGATTTTGCCCAAGC |
| F20 | TAGGGAAGAGAAGGACATATGATTCAAAGCTTTGCCTATCCCTACG |
| F21 | TAGGGAAGAGAAGGACATATGATAGCCAATCATCTCCAGCTCCCGC |
| F22 | TAGGGAAGAGAAGGACATATGATGAGAGGGCGGTCTTTTTAGGCGG |
| F23 | TAGGGAAGAGAAGGACATATGATAAGCGAGTCTTGCCATTGGATAA |
| F24 | TAGGGAAGAGAAGGACATATGATCCTCATTGGGCGGGGCAGCAGAG |
| F25 | TAGGGAAGAGAAGGACATATGATCTCCTCCTTACCCCCCCCTCCCT |
| F26 | TAGGGAAGAGAAGGACATATGATTTCCCGGCCCCCCCCAACCCCCC |
| R01 | TCAAGTGGTCATGTACTAGTCAAAAGGTAAGAACTGAGGCCTCTGC |
| R02 | TCAAGTGGTCATGTACTAGTCAATAAAATGTGAATTCTCCTAAACT |
| R03 | TCAAGTGGTCATGTACTAGTCAAGAGGTCATGTCCTTCAGTATTTG |
| R04 | TCAAGTGGTCATGTACTAGTCAATCTGGCCTCCTCTTTATCCGTGT |
| R05 | TCAAGTGGTCATGTACTAGTCAATAGCTGAGCTCCAGCTTTTTGCA |
| R06 | TCAAGTGGTCATGTACTAGTCAAGCGGATAAGTCACTTCCATTTTT |
| R07 | TCAAGTGGTCATGTACTAGTCAACCCCAGACAAAGGCGCTAACCAC |
| R08 | TCAAGTGGTCATGTACTAGTCAATACTGAGCTGACCAATATGCAGA |
| R09 | TCAAGTGGTCATGTACTAGTCAAGCTGCAAGCCCTGGGAATGCAGG |
| R10 | TCAAGTGGTCATGTACTAGTCAAAGTACCTCTCAAATCTTACCCCC |
| R11 | TCAAGTGGTCATGTACTAGTCAATCGGCGCGCAGTTCCGCATCAAC |
| R12 | TCAAGTGGTCATGTACTAGTCAACAGTCATTAGGTGGGACGCTTTA |
| R13 | TCAAGTGGTCATGTACTAGTCAAGGGAAGAGGGCTATGCCAAGAGG |
| R14 | TCAAGTGGTCATGTACTAGTCAAGTAGGCCTGGCACCGCCTCCTAG |
| R15 | TCAAGTGGTCATGTACTAGTCAAAACTTGGAGTGGCAGAGGAGAAT |
| R16 | TCAAGTGGTCATGTACTAGTCAAAGGAAAGGACGCATATTGACGTG |
| R17 | TCAAGTGGTCATGTACTAGTCAAAAACCAATCAGAAATCAGGCGGG |
| R18 | TCAAGTGGTCATGTACTAGTCAAGCAAAATCCTAGTGGGCGGAGAG |
| R19 | TCAAGTGGTCATGTACTAGTCAAGATAGGCAAAGCTTTGATAGGAA |
| R20 | TCAAGTGGTCATGTACTAGTCAAGCTGGAGATGATTGGCTTGGAAG |
| R21 | TCAAGTGGTCATGTACTAGTCAAAAAAAGACCGCCCTCTCAGTGTT |
| R22 | TCAAGTGGTCATGTACTAGTCAAAATGGCAAGACTCGCTTGCTCTC |
| R23 | TCAAGTGGTCATGTACTAGTCAATGCCCCGCCCAATGAGGGAGGGG |
| R24 | TCAAGTGGTCATGTACTAGTCAAGGGGGGGTAAGGAGGAGGGAGCA |
| R25 | TCAAGTGGTCATGTACTAGTCAATTGGGGGGGGCCGGGAAAAACGC |
| R26 | TCAAGTGGTCATGTACTAGTCAACAGCTGAGGGACAAGCTCAAGGG |
